# The Nutrition Facts of Comfort Foods in the United States: Results from the Census-Matched Eating in America Study

**DOI:** 10.3390/nu18193257

**Published:** 2026-10-02

**Authors:** Dorothy T. Chiu, Jenna R. Cummings, Euridice Martinez-Steele, Alejandra T. Lopez, Jillian Chellis, Juanyi Tan, Katrina M. Smith, Sameeksha Panda, Laura E. Finch, A. Janet Tomiyama

**Affiliations:** 1 Osher Center for Integrative Health, University of California, San Francisco, San Francisco, CA 94115, USA; 2 Department of Psychology, Institute of Population Health, University of Liverpool, Liverpool L69 7ZA, UK; 3 Department of Nutrition, School of Public Health, University of São Paulo, São Paulo 01246-904, Brazil; 4 Center for Epidemiological Studies in Health and Nutrition, University of São Paulo, São Paulo 01246-904, Brazil; 5 Department of Psychology, University of California, Los Angeles, Los Angeles, CA 90095, USA; 6 Department of Chronic Disease Epidemiology, Yale School of Public Health, Yale University, New Haven, CT 06520, USA; 7 The Bridge at NORC, NORC at the University of Chicago, Chicago, IL 60637, USA

**Keywords:** comfort eating, stress eating, American diet, hyperpalatable foods, ultra-processed foods

## Abstract

Background/Objectives: Comfort foods are perceived as energy dense and highly palatable, yet little empirical research documenting the veracity of such perceptions exists. Methods: We analyzed comfort foods for nutrient content, hyperpalatability, ultra-processing, and by demographic factors, hypothesizing poor nutritional profiles (higher fat, sugar, sodium, ultra-processing) and demographic differences in preferences. US census-matched respondents (*N* = 1760) self-identifying as comfort eaters listed their top three “comfort foods” through an online survey assessing comfort eating behavior and demographics (age, gender, US residence region). Trained raters abstracted nutrient contents for nominated foods using public reference databases and compared them against established nutritional recommendations (percent daily values [%DV]). We categorized foods by hyperpalatability and Nova classifications. Summary statistics were calculated overall and by demographic group. Two-sample t- and one-way ANOVA tests evaluated nutritional and demographic group differences. Results: *N* = 300 unique foods/beverages were nominated as top comfort foods, collapsible into *n* = 132 groupings by similarity (e.g., in ingredients, preparation). The top five groupings were chips, ice cream, pizza, chocolate, and cookies. Notable proportions of comfort foods were low (<5%DV) sources of fiber (60.7%), high (>20% DV) sources of saturated fat (29.7%) and sodium (31.7%), hyperpalatable (72.7%) (especially of the “meats/meal” variety (54.3%)), and 100% ultra-processed (42.7%). Slight differences (*p* < 0.05) in preferred foods’ nutrient content, hyperpalatability, and ultra-processing by age, gender, and region emerged. Conclusions: US comfort foods range widely but converge on certain foods. Nutritional quality could broadly improve; demographic differences are minor. These findings enhance the practical understanding of comfort food nutrition and inform efforts towards healthier comfort food selection.

## 1. Introduction

Psychobiologically speaking, comfort eating or eating to comfort oneself is definable as eating in response to negative stimuli (e.g., stress) or affect, and is closely linked to emotional eating (when specific to negative emotions) and stress eating [1]. Epidemiological work estimates the prevalence of comfort eating to a range between 15–46% in general population samples and 47–71% in clinical groups with obesity or eating disorders [1,2,3,4]. “Comfort foods” are commonly assumed to be palatable and energy dense as people often describe feeling drawn to consume fat and sugar and/or salt during negative states [5,6,7,8]. However, rigorous larger scale studies on the nutritional content of comfort foods are lacking.

To date, the empirical research on comfort food preferences remains limited. Previous work, upon which many comfort foods studies have been based [9], documented favorite comfort foods in the US and investigated differences by age and gender [10]. However, it relied on a nonrepresentative sample of US adults and also did not examine nutrition. Moreover, that work itself has spurred controversy [11,12] and may warrant re-examination [13]. Additionally, today’s US food environment has since evolved to comprise more energy-dense foods containing higher than recommended levels of fat, sugar, and sodium. Many of these foods attain “hyperpalatable” (or “hyper-palatable”) status, where combinations of these nutrients result in a synergized boost of hard-to-resist flavor [14], as well as ultra-processed status, where additives and other compounds that serve more cosmetic and/or taste-enhancing purposes are incorporated during industrial food processing [15,16,17,18]. Awareness of the overlap between the attributes of poor nutrient content, hyperpalatability, and ultra-processing is growing [19] as ultra-processed foods frequently contain little to no whole foods and are nutrient poor, energy dense, and highly palatable with elevated levels of fats, added sugars, and/or salt [20]. 

Nutritional epidemiology research has long focused on how such intakes of fats [21], sugar [6,22,23], and sodium [24] generally relate to adverse physical and mental health outcomes, though exact recommendations continue to evolve with research. In the context of comfort eating specifically, connections with abdominal obesity and the obesity epidemic as well as type II and pre-diabetes risk, cardiovascular risk, and stroke have been established [22,25,26,27]. Similarly, concerns are rising about the potential addictive dangers of hyperpalatable foods [28] as well as the higher mortality risks [29,30,31] and morbidities (e.g., excess weight [17], cardiometabolic disease [17], mental disorder [32], and cognitive impairment [33]) associated with ultra-processed foods. Moreover, the recent research has documented correlations between perceived stress and higher ultra-processed food consumption [34]. Thus, this study sought to more comprehensively profile US comfort food nutrition for the broader American population beyond fat, sugar, and salt including more nutrients as well as hyperpalatability and ultra-processing.

Comfort foods were hypothesized to have high levels of energy density, fat, sugar, and salt. Comfort foods were also hypothesized to contain a significant proportion of hyperpalatability and ultra-processing given the ever-increasing availability and intersection of these qualities in today’s food supply [16,17,34]. Lastly, elucidating demographic differences in comfort food preferences by age, gender, and region may facilitate a broader understanding of this complex behavior of great public health nutrition importance. Demographic differences were predicted, although directional hypotheses were not specified with this last aim’s descriptive intent.

## 2. Materials and Methods

### 2.1. Data Source

A national online panel survey called the UCLA Eating in America Study (Eating in America Study) was administered between December 2019 and January 2020. It aimed to assess stress and eating in 2000 adults (the maximum target given the project budget). It recruited a sample of *N* = 2022 adults (≥18 years) via Qualtrics (Provo, UT, USA), which engaged participants matching specific demographic characteristics per US census-based quotas for age, gender, and census/geographic region of residence in addition to race/ethnicity and income. Respondents were asked to respond to the item “What are your top three comfort foods?” The survey also collected demographic information. Gender was self-reported in response to the question “Which best describes your gender?” and answer responses included woman, man, non-binary (*n* = 4), and “I use a different term” (*n* = 1). Gender analyses were limited to men and women given the other categories’ small sample size. Age was self-reported in years but categorized into three groups (18–34 years, 35–54 years, and 55+ years) to replicate Wansink et al.’s initial study [10]. US census region was recorded as Northeast, South, Midwest, or West. A university institutional review board council granted human subjects ethics approval, informed consent was obtained from all participants, and renumeration was provided for participation. Pre-registration of the study focusing on demographic differences is available on Open Science Framework (https://osf.io/85hac, accessed 17 September 2026); the nutritional analyses including hyperpalatability and ultra-processing were added post hoc. This report follows the Strengthening the Reporting of Observational Studies in Epidemiology reporting guideline for nutritional epidemiology studies (STROBE-nut).

### 2.2. Comfort Foods: Identification and Data Abstraction

Two trained research assistants (RAs) independently identified foods and beverages (hereafter “food”) from the free text response data generated by the top comfort foods question. The research team developed a standardized protocol (https://osf.io/c53ab/overview, accessed 17 September 2026) through extensive internal discussions that considered contextual factors such as punctuation usage and line spacing. The respondents’ original answers were preserved to the fullest extent possible to minimize researcher subjectivity. Responses left blank or that were nonsense/not foods/indecipherable (hereafter referred to as incomprehensible, e.g., “Ok”, “nothing”, “chess”) were recorded as such. General phrases (e.g., “anything cheesy”) were also recorded as such and categorized as a nutritionally uncodable food. Combination foods, defined as when ≥2 items were reported together as a comfort food (e.g., “chips and dip”), were recorded per respondent’s words and nutritionally coded to the best extent possible.

### 2.3. Nutritional Information

The trained RAs independently abstracted nutrient information using the USDA’s What’s in the Foods You Eat online search tool (Beltsville, MD, USA; 2019–2020) to search the What We Eat in America (WWEIA) database. This resource contains data on each food’s USDA Food and Nutrient Database for Dietary Studies (FNDDS) 8-digit code as well as calories, protein, carbohydrates, total sugars, total fiber, total fat, saturated fat, and sodium content per 100 g and per its “quantity not specified.” (“Quantity not specified” was taken to approximate the most common/typical serving size for the food.) The RAs first entered each food into the online search tool (verbatim to the participant’s survey response) and then modified search parameters slightly as needed and appropriate to enable the return of results both close to the respondent’s original submission as well as nutritionally codable (e.g., “cereal cold sweet” was adjusted to “cereal”). If ≥1 search result was returned, the RAs chose the most general form available, usually signified by a “not further specified” designation in the database. For items not in the database, the team accessed brand and/or company websites for nutrition information or physical products for actual food labels (e.g., Caramel Milky Way). If these approaches proved unsuccessful, items were deemed nutritionally uncodable (e.g., “Evergood sausage”). The USDA and brand-specific databases were selected for their public online availability and accessibility. The interrater agreement of the nutrient data abstractions by the RAs revealed a discrepancy rate of 0.90% (calculated as [the number of abstracted data points not in agreement between raters]/[the number of data points the raters both abstracted]). All discrepancies were reconciled through the research team’s discussions.

Once the abstracted nutrient data were finalized, the nutrient data for 100 g of the food were applied to the “quantity not specified” weight (hereafter, the amount for a “typical serving”) to proportionally calculate the amount of each nutrient of interest present in a typical serving. Combination foods necessitated further calculations; specifically, we abstracted nutrient information for the “quantity not specified” amount of each component of the combination food (e.g., chips and then dip), summed them, and then calculated nutrient information proportional to 100 g (of the combination food) for use in subsequent analyses.

For each nutrient of interest, the amount present in the typical portion of a food was compared with the nutrient’s recommended daily value (DV) per the US Food and Drug Administration (FDA) to calculate the percent DV (%DV) of the nutrient contained in a typical serving of that food item. The %DV value is a user-friendly public health measure helpful to ascertain if a food is a high or a low source of a nutrient; values are based on a 2000 kcal/day diet [29]. Foods were classified as high, moderate, or low nutrient sources based on the amount of a particular nutrient present in one serving: >20%DV was high, <5%DV was low, and between 5 and 20%DV was moderate [35]. As the FNDDS database only includes data on total sugar (and not added sugar) and because the FDA does not include a DV for total sugars (only added sugars), we used the United Kingdom’s National Health Service Recommended Intake for total sugars (90 g/day for adults) and constructed a “%DV” from this value [36].

### 2.4. Hyperpalatable Food (HPF) Status

Hyperpalatability status was determined by applying Fazzino et al.’s criteria for fat, sugar, sodium, and/or carbohydrate content per serving to a food’s typical serving amount [14]. These criteria identify hyperpalatable foods using key nutrients in various combinations across food categories in a reliable and validated fashion. Fazzino et al.’s work established critical initial documentation of previously “unknown outside of industry” food formulations designed to “maximize palatability and consumption” and override the physiological satiety mechanisms and brain reward neural circuitry critical for sensory-specific satiety. Three hyperpalatable food (HPF) formulations or categories are identified: (1) the high (>20% kcal) fat + high (>20% kcal) sugar “dessert” type; (2) the high (>25% kcal) fat + high (≥0.30% by weight) sodium “meats/meal” type; and (3) the high (>40% kcal) carbohydrate + high (≥0.20% by weight) sodium “bread/carbohydrate” type.

Dichotomous “yes/no” variables were created for each of these HPF categories based on abstracted nutrient data as well as being hyperpalatable in general (i.e., fitting any HPF category).

### 2.5. Ultra-Processed Food (UPF) Status

To determine ultra-processed food (UPF) status, the Nova classification system was applied. It characterizes foods based on the nature, extent, and purpose of their industrial processing into four groups: Group 1, unprocessed or minimally processed foods; Group 2, processed culinary ingredients; Group 3, processed foods; and Group 4, ultra-processed foods [17,18,37]. Specially drawing upon the work of Steele et al. who applied the reference approach to determine the Nova group composition for WWEIA data between 2001 and 2018 [18], each nominated comfort food was linked to their (Steele et al.’s) database repositories of Nova group content through FNDDS codes. This linkage yielded four values representing the proportion of calories corresponding to each Nova group based on total calories [18]. As minor changes are made to FNDDS constituents (not codes) each year, all FNDDS databases between 2001 and 2018 were referenced as needed, starting with the 2017–18 database. Specifically, any comfort foods remaining unmatched after an FNDDS database comparison were matched against progressively older databases, i.e., first 2017–18, then 2015–16, and so on, ending with 2001–02. After all available years were searched, six foods remained: hamburgers, potato skins, and the following four brand-specific items: Lay’s potato chips, Hot Cheetos (including Flaming and puffs), McDonald’s French fries, and Burger King’s Impossible Burger. For these, researchers applied the Nova information for food(s) most similar to these items with respect to ingredients and/or preparation, again prioritizing the 2017–18 FNDDS. For example, potato skins were matched against “potato skins without topping”, “potato skins with cheese”, and “potato skins with cheese and bacon” in the 2017–18 database to obtain its Nova Group 4 content. Since all these options had zero proportion of calories of grams from Nova Group 4, “potato skins” was assigned zero proportion of calories from Group 4. The Hot Cheetos products were matched against “cheese-flavored corn snacks (Cheetos)” in the 2017–18 FNDDS, which had 100% proportion of calories from Group 4. The ultra-processed content of combination foods was calculated by determining the “final” proportion of calories in Nova Group 4 after applying the same proportional approach taken earlier involving the “quantity not specified” measure weights and proportions of Group 4 calories for each constituent food and standardizing it to 100 g. For example, “chips and dip” considers the proportion of calories from Group 4 for the typical measure weights for chips as well as for dip.

A dichotomous “yes/no” UPF status variable was created and defined according to the proportion of calories in Nova processing level Group 4 [18]. This extends previous approaches to define ultra-processed foods as food products comprising ingredients “mostly” of industrial use and/or results of industrial processes, which often necessitated food science expertise and/or subjective assessment [17,38] that can introduce variability and misclassification [18]. To be most conservative, a strictly quantitative approach was taken and UPF status was assigned to foods where the proportion of calories in Group 4 was 100%. This establishes a “minimum floor” with which to position this study’s findings.

### 2.6. Statistical Analyses

Descriptive summary statistics were calculated for: (a) the sample of survey respondents, (b) the collective sets (up to 3) of nominated comfort foods per respondent (e.g., mean nutrient content, hyperpalatability, ultra-processing), and (c) all uniquely nominated and nutritionally codable comfort foods (e.g., frequency of nomination, nutrient content, hyperpalatable status, ultra-processed status). The research team further collapsed foods that were similar (e.g., chips, potato chips, and barbecue chips) into larger “umbrella” groupings (e.g., chips [all types, incl. cheese curls/puffs]) for frequency analyses after extensive research team discussions that considered predominant ingredients and processing (c2). This collapsed list comprises the topmost nominated comfort foods in the US. (Note that not all entries on the collapsed list comprise more than one item; groupings only occurred as needed.) All other analyses for (c) were conducted on uncollapsed foods, as nutrient and Nova information was available at this level. Demographic group differences were tested via two sample *t*-tests and one-way ANOVAs for continuous variables and chi-squared tests for categorical variables. Additionally, supplemental post hoc analyses explored associations between nutrient content and having hyperpalatable and/or ultra-processed statuses. Modified Poisson regression with a robust estimator of variance was used, as this method is helpful when approximating relative risk ratios for binary data with outcomes >10% [39,40,41]. Statistical significance was set at α = 0.05 and analyses utilized Stata15 SE (College Station, TX, USA).

## 3. Results

### 3.1. Respondent Characteristics

Descriptive demographics are presented in Table 1 along with summary nutritional information for nominated comfort foods by respondent. Of 2022 survey respondents, *n* = 1760 had analyzable comfort food data, nominating up to three comfort foods. Of the *n* = 1760, *n* = 1228 (69.8%) provided ≥1 nutritionally codable comfort food (see Supplemental Figure S1 for study respondent flowchart). Those identifying as men (*X*^2^ = 8.05, *p* = 0.005) and age 55+ (*X*^2^ = 9.84, *p* = 0.007) were less likely to nominate ≥1 nutritionally codable comfort food compared with those identifying as women and younger persons. No differences in nomination were observed by US region of residence (*X*^2^ = 3.15, *p* = 0.37). On average, amongst respondents specifying ≥1 nutritionally codable comfort food, preferred comfort foods contained high levels of percent calories from fat (40.1%) and a large proportion of calories from Nova Group 4 (0.78 out of 1.0).

### 3.2. Comfort Foods

#### 3.2.1. Top Comfort Foods

Out of 5280 possible nomination slots for comfort foods, 26.8% (*n* = 1417) were left blank, 3.7% (*n* = 193) were incomprehensible (e.g., “juke”), and 8.1% (*n* = 429) were nutritionally uncodable (e.g., “salty snacks”, “anything”). The remaining 61.4% (*n* = 3241) of responses were nutritionally codable and had abstractable nutritional information (e.g., “potato chips”, “Cheetos”, or “Takis”), from which a total of 300 unique nutritionally disparate foods were extracted. (This latter number is smaller as foods were nominated more than once.) These 300 were further collapsed into 132 comfort foods based on similarity (e.g., “potato chips”, “Cheetos”, and “Takis” were collapsed into the same group of “Chips [all types]”). More examples can be found in Supplemental Table S1, which presents the top 10 collapsed foods along with their constituents in order of nomination prevalence.

Table 2 displays the top 50 most nominated foods by number of mentions, after collapsing similar foods. The top 15 comfort foods in the US were chips, ice cream, pizza, chocolate, cookies, candy, burgers, cake, pasta, bread, cheese, macaroni and cheese, fries, soda, and chicken/chicken dishes. Beverages figured prominently: in addition to soda (#14) and coffee (#36), milk, wine, beer, and water/ice tied in 65th place. Foods traditionally perceived as healthful were also frequently nominated: nuts (#20), fruits (#24), potatoes (#25), eggs (#38), vegetables (#38), and salad (#44) were observed in the top 50. Supplemental Table S2 displays the remaining top comfort foods after the top 50.

#### 3.2.2. Nutritional Facts

Table 3 displays summary statistics for all the nutritionally unique comfort foods (*n* = 300). Summary statistics were not weighted and calculated without regard to nomination frequency. On average, US comfort foods contained about 223.1 kcal (SD = 148.2) per serving, fell into the moderate range for all nutrients of interest (i.e., means were all within the 5–20%DV range), and had 18.3% (SD = 24.1) calories from sugar, 39.2% (SD = 20.8) calories from fat, and a proportion of 0.57 (SD = 0.44) calories from ultra-processed Nova Group 4. Table 4 summarizes prevalences related to low, medium, and high sources of each nutrient of interest as well as hyperpalatable and ultra-processed status. The majority of top comfort foods fit the criteria for being moderate sources of total fat (50.0%, *n* = 150 foods) and carbohydrate (60.0%, *n* = 180) and low sources of total sugar (56.0%, *n* = 168) and fiber (60.7%, *n* = 182). Substantial proportions of comfort foods were moderate sources of protein (41.3%, *n* = 124), saturated fat (43.3%, *n* = 130), and sodium (46.3%, *n* = 139). In comparison, the proportions of comfort foods that were high sources of total fat were 23.3% (*n* = 70), 29.7% (*n* = 89) for saturated fat, 31.7% (*n* = 95) for sodium, and 17.0% (*n* = 51) for total sugar. (Supplemental Table S1 also visually highlights low and high nutrient sources among the top 10 comfort foods and their constituents.)

#### 3.2.3. Hyperpalatability and Nova Processing

For hyperpalatability, 72.7% (*n* = 218) of comfort foods fit Fazzino’s [14] criteria. The biggest proportion consisted of the high fat and high sodium “meats/meal” type (54.3%, *n* = 163), followed by the high fat and high sugar “dessert” type (21.3%, *n* = 64), and lastly followed by the high carbohydrate and high sodium “bread/carbohydrate” (“bread/carb”) type (11.0%, *n* = 33) (Table 4). Regarding food processing, 42.7% (*n* = 128) of comfort foods were considered ultra-processed (i.e., 100% calories attributable to Nova Group 4). Thirty-six percent (*n* = 108) of comfort foods were both hyperpalatable (any type) and ultra-processed. (Supplemental Table S1 reports on hyperpalatability and ultra-processing status.) Pasta (#9) was the only top 10 comfort food without a hyperpalatable or 100% ultra-processed distinction. All constituent items of the top four comfort foods (collapsed) were hyperpalatable and 100% ultra-processed. Supplemental Table S3 displays the frequency of overlap between various nutrient, hyperpalatability, and ultra-processed indicators, and Supplemental Table S4 further displays the estimated likelihoods of being an HPF or UPF associated with nutrients.

### 3.3. Demographic Differences

#### 3.3.1. By Gender

Men’s and women’s top comfort foods were statistically different on all nutritional indicators: nutrient content, hyperpalatability, and food processing (Table 5A,B, Supplemental Table S5). Using daily values as a benchmark, average nutrient differences were equivalent to 0.7%DV (total carbohydrate) to 2.5%DV (saturated fat) and 3.9%DV (sodium) to 6.4%DV (total protein). Men’s comfort foods were ~33 kcal higher (95% CI: 19.5, 46.7) per serving than women’s, and also higher in all other nutrients assessed including protein (δ = 3.2 g, 95% CI: 2.5, 4.0), saturated fat (δ = 0.5 g, 95% CI: 0.2, 0.8), fiber (δ = 0.3 g, 95% CI: 0.1, 0.4), and sodium (δ = 90.4 mg, 95% CI: 56.6, 124.1). Women’s top comfort foods were higher than men’s only for total sugar (δ = 1.2 g, 95% CI: 0.4, 2.0). 

#### 3.3.2. By Age

The primary differences in top comfort foods by age were between 18–34-year-olds and those 55+ on nutrient content; comfort foods of 18–34-year-olds and 35–54-year-olds did not statistically differ on those criteria. Compared with those age 18–34, those 55+ nominated foods were significantly lower in calories (δ =40.8 kcal, 95% CI: 23.9, 57.7), protein (δ = 2.5 g, 95% CI: 1.5, 3.4), fat (δ = 0.9 g, 95% CI: 0.2, 1.7), carbohydrate (δ = 5.6 g, 95% CI: 3.6, 7.6), dietary fiber (δ = 0.4 g, 95% CI: 0.3, 0.6), and sodium (δ = 95.0 mg, 95% CI: 53.0, 137.0) per serving; foods did not differ on saturated fat or total sugar content. Average nutrient differences were similar to the above, ranging between being equivalent to 1.2%DV (total fat) to 4.1%DV (sodium) to 5%DV (total protein).

#### 3.3.3. By Region

Statistically significant nutritional differences were observed by region. Northeastern US residents’ top comfort foods were higher in calories, protein, total carbohydrate, dietary fiber, and sodium than those of southern and midwestern US residents. Southerners’ comfort foods also had significantly lower saturated fat content versus northeasterners. Westerners’ comfort foods were only lower in total carbohydrates and dietary fiber than northeasterners. There were no statistically significant differences for other nutrients by region. Like observed differences between gender and age, regional differences had magnitudes of difference 4% DV and below (Table 5A,B).

Minor, but significant, differences in hyperpalatability and ultra-processing were found by gender, age, and region (Supplemental Table S5).

## 4. Discussion

To the authors’ knowledge, this study presents the first comprehensive nutritional analysis of top comfort foods nominated amongst the general American population, encompassing nutrient content as well as hyperpalatability and ultra-processing. A wide variety of foods and drinks were elicited as top comfort foods, but the topmost items were chips, ice cream, pizza, chocolate, and cookies. Interestingly, more healthful foods (i.e., nuts, fruits, eggs, vegetables, salad) and beverages (i.e., coffee) also appeared in the top 50. On average, top comfort foods contained slightly more than 200 kcal or 10% of a day’s recommended caloric intake for a common adult 2000 kcal/day diet per serving. They were moderate sources of macronutrients (e.g., total fat, carbohydrate, protein), low sources of fiber, and moderate sources of saturated fat and sodium. Notable but smaller proportions of top comfort foods also fit the criteria for being high sources of total and saturated fat, sodium, and total sugar. A majority (~73%) of comfort foods were hyperpalatable, with most fitting the criteria for the high fat, high sodium “meats/meal” HPF type, followed by the high fat, high sugar “dessert” type. Nominated comfort foods contained a substantial proportion (~78%) of ultra-processed content on average, and a considerable (~40%) proportion met our criteria of being an ultra-processed food with 100% calories attributable to Nova Group 4.

Demographic differences in comfort food preferences were observed but slight—generally on the scale of <5%DV for their respective nutrients. Men’s top comfort foods contained greater amounts of all nutrients of interest (i.e., calories, macronutrients, saturated fat, sodium) versus women’s, except for total sugar, of which women’s contained more. Men’s comfort foods also included more of the “meats/meal” HPF type, while women’s included more of the “dessert” and “bread/carbohydrate” types and more 100% ultra-processed items. By age, the main differences were observed between 18–34-year-olds and those age 55+. Average saturated fat and total sugar content did not significantly differ with age, though means for all other nutrients were lower in those age 55+. Regionally, northeastern and western residents’ top comfort foods were more similar to each other versus southern and midwestern US residents’ foods.

These findings generally coincide with other comfort food studies, though there are some deviations. Compared with Wansink’s original study of comfort foods, as well as a more recent study by Betancourt-Nuñez et al., nearly all the top 10 comfort foods identified here were among the top foods identified in the other works [10,42]. Wansink’s group also identified vegetables/salads and soup amongst comfort food favorites, though these items appeared lower in our study (as #38/#44 and #20, respectively) [10]. Similar to this study, Betancourt-Nuñez et al. also identified a greater presence of higher fat and sugar and lower fiber foods with emotional eating [42]. Regarding demographic differences, the significant ones observed in this study were small. Men were found to prefer comfort foods of the heartier meal-related variety, as Wansink also reported [10]. Women were observed to nominate more foods that were higher in sugar and of the “dessert” variety, akin to the preferences for candy, chocolate, and ice cream that Wansink also observed [10]. Indeed, gendered food preferences have been substantiated in the literature, illustrating the power of gender role conformity and identity in eating behaviors [43]. However, while Wansink observed older respondents to prefer more meal-related comfort foods than their younger counterparts [10], no significant differences by age, save for a slight preference for dessert-type comfort foods among those 55+, were observed here. This could reflect recent trends reflecting less home cooking amongst certain groups [44]. Notably, this study’s data were collected in 2019–2020 prior to the COVID-19 pandemic, a globally traumatic event that may have delivered its own heightened level of stress eating [45,46]. However, Gemesi et al., who observed similar comfort foods in Germany post-COVID [47], suggest that comfort foods do not evolve very much or quickly.

This study’s quantification and profile of the nutrient contents of comfort foods supports the idea that foods looked to during stressful times are often higher in carbohydrates, sugar, salt, and/or fat [6,22]. Per stress physiology, when an individual is met with a stressor, the hypothalamic–pituitary–adrenal (HPA) axis (and sympathetic–adrenal–medullary axis) is triggered to mediate the body’s physiological response. Ensuing actions serve to increase the availability of fuel substrates (e.g., free fatty acids, glucose, amino acids) so an organism can optimally mobilize to escape unfavorable situations. Elevated levels of cortisol, the primary hormone of HPA activity, engenders a course of metabolic, cognitive, behavioral, and neuroendocrine changes (including potentiation of reward processing pathways), directly and indirectly promoting eating [6,48,49]. Notably, in non-human animal models, cortisol appears to intensify drives to consume fat and carbohydrates and/or sugar, a behavior that can relieve feelings associated with stress when ingested and end acute stress responses by tamping down cortisol release via negative feedback loops [1]. While this cascade can be helpful in the short term and was evolutionarily key [50,51], it can prove detrimental for long-term health, especially in the contexts of increased stress and readily available and access to tasty food, such as in todays’ food and social environment [50,52].

This study also offers previously undocumented nutritional context by comprehensively examining caloric and other nutrient composition while additionally considering federal nutritional recommendations (%DVs) and linking such to hyperpalatability and ultra-processing. These attributes are of increasing importance to study in tandem. There are notable nuances present in comfort food nutrition. For example, foods that are veritably “low sources” of concerning nutrients (e.g., total/saturated fat, sugar, sodium) by DVs were also among the top comfort foods named. Comfort foods do not necessarily present as concerning for all “problem” nutrients simultaneously, e.g., ice cream is a high source of both saturated fat and sugar and a low source in dietary fiber while being a low source of sodium. Further, some “meats/meal” HPF types were high sources of total fat and sodium, but naturally low sources of sugar. Interestingly, it was observed that fewer high fat and high sugar foods were flagged as such based on %DVs and that more foods fell into the high fat, high sugar “dessert” HPF type category. This highlights the unique values and limitations of these various indicators: %DVs employ a blunter demarcation of foods using the 5% and 20% marks and consider nutrients one at a time while HPF classifications, which consider more than one nutrient at once, do so more stringently and at different levels.

Many of the public’s top comfort foods, especially those in the top 10, were classifiable as hyperpalatable and ultra-processed. The surprising outcome that less than half of all nominated foods fit this study’s criteria for being ultra-processed is likely related to its conservative definition of UPFs. Indeed, on (unweighted) average, a significant proportion of calories of nominated comfort foods were attributable to ultra-processed sources. Additionally, the hyperpalatability findings point to the importance of considering psychosocial aspects of comfort eating. For instance, most comfort foods that were hyperpalatable foods fit the “meats/meal” type, which could reflect foods more characteristic of home cooking. Home-cooked foods evoke social and emotional memories and have been associated with love, care, and nostalgia [53].

It is vital to objectively characterize comfort foods for public health nutrition purposes. While we did not assess whether respondents were undergoing stress while answering this survey, these are foods that were reported as favorites during times of needing comfort. The American Psychological Association’s annual *Stress in America* assessments report persistent feelings of heightened moderate stress across the American population [54,55]. Comfort eating remains a common coping mechanism and, therefore, a potent public health risk factor and behavioral intervention target. The generally small magnitude of demographic differences in comfort food preferences, at least when assessed on person-level means, suggests that targeting population subgroups for intervention may have less utility than targeting top comfort foods as a whole. Still, depending on actual levels of consumption, even small differences between groups could compound significantly, especially over the life course. Thus, considerations of comfort eating may be important for stress and eating studies, particularly those involving more underserved or historically marginalized populations with less resources for stress resilience. It is promising that some work has shown that people would opt for healthier comfort food substitutes if presented a viable alternative [47] and that experimental studies have found no differences in stress reduction benefits of healthy versus unhealthy comfort foods [56]. As was observed, comfort foods do include some nutritious food items, and it may be possible to thoughtfully promote healthier comfort eating. While some health advocates may question the idea of intervening on comfort eating versus pushing alternative stress coping strategies altogether, this would be remiss as the formidable 15–46% prevalence of comfort eating amongst the general population [1,2,4] makes such an approach short-sighted.

Strengths of this study include: (1) the use of standardized protocols to abstract comfort foods from free text responses and (2) the reference and utilization of robust national and/or established nutritional databases, resources, and systems (i.e., for HPFs, Nova) for nutritional content analyses. The large US census-matched sample (matched on gender, race/ethnicity, age, income, and census region) also allows us to expect that these results can be reasonably generalized to the American population with some constraints [57]. They are less generalizable than a random sample of the United States and not generalizable to children, incarcerated adults, and others not reachable by the Prolific platform. Results will also not generalize to those of non-binary gender, who were excluded given the small sample size, nor to countries other than the US. These results were also obtained before the surge in glucagon-like peptide-1 (GLP-1) and other similar medications that have been shown to substantially affect eating behaviors. The nutrition facts of favorite comfort foods may have shifted in the GLP-1 era or shift as more of the population takes these medications.

Other limitations of this study include the fact that comfort foods were self-reported and nominated in response to one item asking about top comfort foods. Another weakness was that nominations were limited to the level of detail provided by respondents, so not all factors that affect nutritional content (e.g., food preparation) could be accounted for. Indeed, one challenge of determining Nova classification for UPFs is discerning sufficient detail regarding sources and preparation of ingredients [18]. Still, nutrient values in the FNDDS database reflect averages for generic versions of food items [58]. Relatedly, the classification systems of HPFs and UPFs used here should also be considered with a grain of salt. Fazzino et al. themselves caution that the HPF criteria they offer should be taken as a starting point for more research, as the extent to which the identified nutrient combinations are sensitive to influencing palatability amongst the American population and in the US food environment have yet to be fully examined and could be at risk for error [14]. Also, the pervasiveness of ultra-processed foods amongst top US comfort foods is likely underestimated given that having 100% of calories attributable to Nova Group 4 sources was used to define UPF here. Additionally, nominations do not translate to actual intake, or intakes under stress at that. It was also not possible to know how often and/or how much nominated comfort foods were eaten by respondents, how much stress participants might have been feeling at the time of completing the survey, or how comfort foods fit into one’s overall diet and/or relate to one’s health. Rather, these data are strictly representative of popularity and future work is needed to better characterize comfort eating in overall diet and assess health risks and/or “harmful” doses. On a similar note, findings were based on averages that were unweighted and did not consider frequency of nomination; weighted analyses could be informative in the future. Data were also limited by the nutrients available for abstraction. For example, this study reports on total sugar even though added sugar would have more public health relevance. (This approach may also partially account for our findings regarding high and low sources of sugar in comfort foods.) Furthermore, the weight or volume-based procedures we used to calculate nutrient density may not translate well to beverages as well as it does for foods. Similarly, combination foods used 100 g as a typical serving size for related analyses (e.g., %DV), which may overestimate or underestimate real composition. However, this approach provided an unbiased, uniform way to include data representative of surprisingly prevalent comfort foods and practices and preserve them for analyses. Future work can improve on this approach.

Lastly, it is important to recognize that comfort foods offer comfort through a variety of means beyond the psychobiological, for example, by acting on feelings of nostalgia, indulgence, convenience, and providing physical comfort [59]. Comfort food preferences stem from their palatability and potential to induce both physiological as well as psychological relief when consumed, though this distinction often appears obfuscated in the literature [22,60]. Here, despite soliciting “comfort foods” in the context of a comfort eating survey, we recognize that actual comfort eating practices may not necessarily comprise solely comfort foods or what one may perceive as their “comfort foods”. Disentangling these details was not possible in these analyses, but deeper examination would be warranted. For example, distinguishing between the impetus and origins for preferences can be critical for interventional strategies. Food choice research cites culture, ritual, tradition, norms, identity, and behavioral patterns as important determinants that reflect the socioecological spheres that people occupy [61,62]. For example, foods that are commonly available in a region are more often reported as comfort foods, including both traditional and more modern foods or cuisines brought in by common immigrant groups [60]. Thus, comfort eating behavioral change approaches would do well to vary with respect to individuals, populations, and their contexts. This is indeed evidenced by the wide range of responses we received to the top comfort foods question from blank to specific food combinations (e.g., “beef chili with pickled jalapeños”) to uncodable (e.g., the vague “anything” or the brand-specific but not item-specific “Evergood sausage”). Continued work in this area will be beneficial and enlightening.

## 5. Conclusions

Top US adult comfort foods vary widely but generally converge on a common list of items. The nutritional quality of these foods could broadly improve, especially as many items are hyperpalatable and 100% ultra-processed. Focusing on popular comfort foods in general rather than on demographic differences may be pragmatic as the latter are slight, perhaps indicative of the pervasiveness of today’s US food system. These findings establish a compendium of comfort food nutrition and can support and inform efforts towards more healthful comfort food selection in response to negative affect and stress.

## Figures and Tables

**Table 1:** Summary statistics of the survey respondent sample (*N* = 1760).

	Mean (SD) or *n* (%) ^a^
**Respondent Characteristics**	
Gender	
Man	865 (49.2%)
Woman	890 (50.6%)
Other	5 (0.3%)
Age	
18–34 years old	470 (26.7%)
35–54 years old	596 (33.9%)
55+ years old	694 (39.4%)
US Region of Residence	
Midwest	400 (22.7%)
Northeast	303 (17.2%)
South	643 (36.5%)
West	414 (23.5%)
**Nutrient Content**—mean ^b^ of nominated food(s) per respondent	
Macronutrients, per serving	
Calories (kcal)	243.4 (122.4)
Protein (g)	7.9 (7.2)
Fat (g)	11.1 (5.7)
Carbohydrate (g)	28.3 (14.5)
Saturated Fat (g)	4.2 (2.7)
Sugar (g)	9.5 (7.0)
Dietary Fiber (g)	1.6 (1.1)
Sodium (mg)	349.8 (303.8)
**Hyperpalatable Food-related Characteristics**—mean ^b^ content of nominated food(s) per respondent	
% Calories from nutrient	
Carbohydrate	21.2 (10.9)
Sugar	20.5 (16.4)
Fat	40.1 (12.9)
% Sodium by Weight	0.35 (0.16)
**Nova Food Processing Characteristics**—mean ^b^ processed content of nominated food(s) per respondent	
Proportion (%) of Calories per Nova Group	
Nova Group 1—unprocessed/minimally processed	0.15 (0.23)
Nova Group 2—processed culinary ingredients	0.02 (0.07)
Nova Group 3—processed foods	0.05 (0.13)
Nova Group 4—ultra-processed foods	0.78 (0.28)

^a^ Including missing. ^b^ Amongst respondents who nominated at least 1 nutritionally codable comfort food (*n* = 1228).

**Table 2:** Comfort food question responses and Top 50 ^a^ comfort foods ^b^ (collapsed) in the US: individual and cumulative prevalences ^c^.

		Prevalence *n* (%)	Cumulative Prevalence (%)
**For all responses** (*N* = 5280):			
	Response Type		
	Blank	1417 (26.8%)	--
	Incomprehensible	193 (3.7%)	--
	Nutritionally Uncodable Foods	429 (8.1%)	--
	Nutritionally Codable Foods *	3241 (61.4%)	--
* Amongst nutritionally codable foods only (*n* = 3241):			
Rank	Food		
1	Chips (all types, incl. cheese curls/puffs)	425 (13.1%)	13.1%
2	Ice cream (all, incl. dairy free)	324 (10.0%)	23.1%
3	Pizza (all kinds, incl. pizza rolls)	274 (8.5%)	31.6%
4	Chocolate (all, incl. dark)	260 (8.0%)	39.6%
5	Cookies (all, incl. cookie dough)	186 (5.7%)	45.3%
6	Candy (all non-chocolate)	173 (5.3%)	50.7%
7	Burgers (incl. veggie)	104 (3.2%)	53.9%
8	Cake (incl. cupcakes)	92 (2.8%)	56.7%
9	Pasta (incl. noodles unspecified)	88 (2.7%)	59.4%
10	Bread (incl. with spread)	82 (2.5%)	62.0%
11	Cheese (all)	78 (2.4%)	64.4%
12	Macaroni and cheese	63 (1.9%)	66.3%
12	Fries (incl. with topping)	63 (1.9%)	68.3%
14	Soda (not incl. diet)	59 (1.8%)	70.1%
15	Chicken/chicken dishes (not incl. fried or wings)	41 (1.3%)	71.3%
16	Crackers (all types)	40 (1.2%)	72.6%
17	Popcorn	39 (1.2%)	73.8%
17	Fried chicken (incl. fried chicken dishes)	39 (1.2%)	75.0%
19	Sandwiches (all types)	36 (1.1%)	76.1%
20	Tacos	31 (1.0%)	77.0%
20	Soups/stews (incl. chili)	31 (1.0%)	78.0%
20	Nuts (all types)	31 (1.0%)	79.0%
20	Rice/rice dishes	31 (1.0%)	79.9%
24	Fruits (unspecified)	30 (0.9%)	80.8%
25	Steak (incl. chicken fried)	27 (0.8%)	81.7%
25	Cereal	27 (0.8%)	82.5%
25	Potatoes (not incl. mashed or fried)	27 (0.8%)	83.3%
28	Spaghetti (incl. spaghetti w/ meatballs)	26 (0.8%)	84.1%
29	Donuts (all types)	25 (0.8%)	84.9%
30	Pies (all types)	24 (0.7%)	85.7%
30	Mashed potatoes (incl. with or without gravy)	24 (0.7%)	86.4%
32	Peanut butter	21 (0.7%)	87.0%
33	Meat/meat dishes (unspecified, e.g., BBQ)	20 (0.6%)	87.7%
34	Ground meat dishes (incl. meatloaf)	16 (0.5%)	88.2%
34	Dessert breads (cinnamon rolls, pastries, NOT muffins)	16 (0.5%)	88.7%
36	Coffee	14 (0.4%)	89.1%
37	Chicken wings (incl. hot wings)	13 (0.4%)	89.5%
38	Eggs	11 (0.3%)	89.8%
38	Pretzels (incl. soft)	11 (0.3%)	90.2%
38	Vegetables (unspecified)	11 (0.3%)	90.5%
41	Ramen (incl. instant)	10 (0.3%)	90.8%
42	Nachos	9 (0.3%)	91.1%
42	Dip (incl. cheese, onion)	9 (0.3%)	91.4%
44	Brownies	8 (0.3%)	91.6%
44	Chips and salsa	8 (0.3%)	91.9%
44	Chips and dip	8 (0.3%)	92.1%
44	Salad (including with dressing)	8 (0.3%)	92.4%
48	Unique, specific “combination snacks” (e.g., tortilla + peanut butter)	7 (0.2%)	92.6%
48	Hot dogs (incl. chili, corn)	7 (0.2%)	92.8%
48	Fish/fish dishes (non-shellfish)	7 (0.2%)	93.0%
48	Cheesecake/cheesecake combinations	7 (0.2%)	93.2%

^a^ Including ties. ^b^ As similar foods were grouped together where possible from research team discussions, this list could be better considered as a list of top comfort food *groupings* in the United States. A grouping can comprise many nominations of a type of food or beverage, or just one (e.g., for chips, many items that were nutritionally disparate were collapsible into a “chip” category given similarities perceived by the research team, but for pierogies, there was only 1 nomination and no other food deemed similar so it comprised its own grouping). ^c^ This table is organized into 2 sections. * denotes amongst nutritionally codable-d foods: The first section reports on the responses to the “what are your top 3 comfort foods” question by prevalence of response type. The latter section reports on the top food groupings found amongst the nutritionally codable foods responses.

**Table 3:** Summary of nutritional attributes per serving for unique top comfort foods (*n* = 300, uncollapsed) in the US.

Characteristic	Mean (SD)
Macronutrients	
Calories (kcal)	223.1 (148.2)
Protein (g) [2.5–10 g] ^a^	8.0 (8.6)
Total Fat (g) [3.9–15.6 g] ^a^	10.5 (9.1)
Carbohydrate (g) [13.75–55 g] ^a^	24.4 (19.5)
Sugar (g) [4.5–18 g] ^b^	8.4 (12.2)
Dietary Fiber (g) [1.4–5.6 g] ^a^	1.5 (1.5)
Saturated Fat (g) [1–4 g] ^a^	3.4 (3.7)
Sodium [115–460] ^a^	362.8 (3.7)
Hyperpalatable Food-Related Characteristics	
% Calories from Nutrient	
Carbohydrate	16.3 (16.1)
Sugar	18.3 (24.1)
Fat	39.2 (20.8)
% Sodium by Weight	0.39 (0.29)
Food Processing Characteristics—Proportion of Calories per Nova Group	
Nova Group 1—unprocessed/minimally processed	0.27 (0.38)
Nova Group 2—processed culinary ingredients	0.06 (0.14)
Nova Group 3—processed foods	0.09 (0.25)
Nova Group 4—ultra-processed foods	0.57 (0.44)

^a^ This range is provided for reference and represents the values considered to be a moderate source of a nutrient, where moderate is defined as foods providing between 5 and 20% daily values of a recommended daily intake per serving calculated on a 2000 kcal/day diet. ^b^ Constructed using the United Kingdom’s National Health Service recommended intake for total sugars.

**Table 4:** Prevalence of low, moderate, and high nutrient sources, hyperpalatable food type and ultra-processed status amongst unique top comfort foods (*n* = 300, uncollapsed) in the US.

	Amongst All Comfort Foods (*n* = 300)—*n* (%)
**Nutrient Sources, by % Daily Values (DVs) ^a^**	
Protein	
Low	94 (31.3%)
Moderate	124 (41.3%)
High	82 (27.3%)
Total Fat	
Low	80 (26.7%)
Moderate	150 (50.0%)
High	70 (23.3%)
Total Carbohydrate	
Low	104 (34.7%)
Moderate	180 (60.0%)
High	16 (5.3%)
Total Sugar ^b^	
Low	168 (56.0%)
Moderate	81 (27.0%)
High	51 (17.0%)
Dietary Fiber	
Low	182 (60.7%)
Moderate	111 (37.0%)
High	7 (2.3%)
Saturated Fat	
Low	81 (27.0%)
Moderate	130 (43.3%)
High	89 (29.7%)
Sodium	
Low	66 (22.0%)
Moderate	139 (46.3%)
High	95 (31.7%)
**Hyperpalatable Food**	
High Fat, High Sugar “dessert” Type	64 (21.3%)
High Fat, High Sodium “meats/meal” Type	163 (54.3%)
High Carbohydrate, High Sodium “bread/carb” Type	33 (11.0%)
Any Hyperpalatable Food Type	218 (72.7%)
Ultra-Processed Food ^c^	128 (42.7%)
**Hyperpalatable and Ultra-Processed Foods**	
Any Type of Hyperpalatable Food + Ultra-Processed ^c^	108 (36.0%)
High Fat, High Sugar “dessert” Type + Ultra-Processed ^c^	70 (23.3%)
High Fat, High Sodium “meats/meal” Type + Ultra-Processed ^c^	44 (14.7%)
High Carbohydrate, High Sodium “bread/carb” Type + Ultra-Processed ^c^	31 (10.3%)

^a^ Where high, moderate, and low nutrient sources are calculated based on amount per serving. ^b^ Constructed using the United Kingdom’s National Health Service recommended intake for total sugars. ^c^ Where the proportion of calories from Nova Group 4 is 1.0 or 100%.

**Table 5:** (**A**) Comparisons of person-level mean nutritional content per serving of top comfort foods by gender, age, and US region of residence. Part 1: total calories and macronutrients: protein, fat, and carbohydrates. (**B**) Comparisons of person-level mean nutritional content per serving of top comfort foods by gender, age, and US region of residence. Part 2: saturated fat, sugars, fiber, and sodium.

(**A**)													
			**Total** **Calories (kcal)**			**Total** **Protein (g)**			**Total** **Fat (g)**			**Total** **Carbohydrate (g)**	
Demographic Characteristic	*n*	Mean (SD) per serving	Difference (95% CI) *%* ^a^	*p*-value	Mean (SD) per serving	Difference (95% CI) *%DV* ^b^	*p*-value	Mean (SD) per serving	Difference (95% CI) *%DV* ^c^	*p*-value	Mean (SD) per serving	Difference (95% CI) *%DV* ^d^	*p*-value
Gender													
Woman (ref)	649	227.9 (111.8)			6.4 (6.2)			10.5 (5.4)			27.4 (13.2)		
Man	577	261.0 (131.3)	**33.1** **(19.5, 46.7)** ***1.6%***	**<0.001**	9.6 (7.8)	**3.2** **(2.5, 4.0)** ***6.4%DV***	**<0.001**	11.9 (5.9)	**1.4** **(0.8, 2.0)** ***1.8%DV***	**<0.001**	29.3 (15.9)	**1.8** **(0.2, 3.5)** ***0.7%DV***	**0.03**
Age													
18–34 yo (ref)	349	262.2 (132.4)			8.8 (7.6)			11.6 (5.8)			31.5 (15.6)		
35–54 yo	422	251.7 (128.4)	−10.5 (−27.7, 6.7) *−0.5%*	0.23	8.9 (7.7)	0.1 (−0.9, 1.1 *0.2%DV*	0.86	11.2 (6.0)	−0.4 (−1.2, 0.4) −*0.5%DV*	0.32	29.1 (15.0)	−1.9 (−4.0, 0.1) −*0.7%DV*	0.06
55+ yo	457	221.4 (104.3)	**−40.8** **(−57.7, −23.9)** **−*2.0%***	**<0.001**	6.3 (5.8)	**−2.5** **(−3.4, −1.5)** **−*5.0%DV***	**<0.001**	10.7 (5.3)	**−0.9** **(−1.7, −0.2)** **−*1.2%DV***	**0.02**	25.4 (12.6)	**−5.6** **(−7.6, −3.6)** **−*2.0%DV***	**<0.001**
Region													
Northeast (ref)	211	267.7 (132.6)			8.9 (7.4)			11.6 (5.6)			32.3 (16.4)		
South	439	234.4 (118.3)	**−33.3** **(−53.4, −13.3)** −***1.7%***	**0.001**	7.5 (7.1)	**−1.4** **(−2.6, −0.2)** −***2.8%DV***	**0.02**	10.7 (5.5)	−0.9 (−1.8, 0.1) −*1.2%DV*	0.07	27.2 (13.7)	**−5.1** **(−7.4, −2.7)** **−*1.9%DV***	**<0.001**
Midwest	293	236.2 (110.9)	**−31.6** **(−53.2, −10.0)** −***1.6%***	**0.004**	7.4 (6.5)	**−1.5** **(−2.8, −0.3)** −***3.0%DV***	**0.02**	11.1 (5.7)	−0.6 (−1.6, 0.5) −*0.8%DV*	0.28	27.2 (13.5)	**−5.1** **(−7.7, −2.5)** **−*1.9%DV***	**<0.001**
West	285	246.7 (129.7)	−21.1 (−42.8, 0.7) *−1.1%*	0.06	8.2 (7.7)	−0.7 (−2.0, 0.6) −*1.4%DV*	0.27	11.4 (6.0)	−0.2 (−1.2, 0.8) −*0.3%DV*	0.67	28.2 (15.0)	**−4.1** **(−6.7, −1.5)** **−*1.5%DV***	**0.002**
(**B**)													
			**Saturated Fat (g)**			**Total Sugars (g)**			**Dietary Fiber (g)**			**Sodium (mg)**	
Demographic Characteristic	*n*	Mean (SD) per serving	Difference (95% CI) *%DV* ^a^	*p*-value	Mean (SD) per serving	Difference (95% CI) *%“DV”* ^b^	*p*-value	Mean (SD) per serving	Difference (95% CI) *%DV* ^c^	*p*-value	Mean (SD) per serving	Difference (95% CI) *%DV* ^d^	*p*-value
Gender													
Woman (ref)	649	4.0 (2.5)			10.1 (6.9)			1.5 (1.0)			307.6 (279.9)		
Man	577	4.5 (2.9)	**0.5** **(0.2, 0.8)** ***2.5%DV***	**0.001**	8.9 (7.0)	**−1.2** **(−2.0, −0.4)** ***−1.3%“DV”***	**0.002**	1.7 (1.2)	**0.3** **(0.1, 0.4)** ***1.1%DV***	**0.0001**	398.0 (322.5)	**90.4** **(56.6, 124.1)** ***3.9%DV***	**<0.001**
Age													
18–34 yo (ref)	349	4.4 (2.8)			9.8 (6.9)			1.8 (1.3)			389.8 (332.22)		
35–54 yo	422	4.3 (2.8)	−0.1 (−0.5, 0.3) *−0.5%DV*	0.63	9.0 (6.8)	−0.8 (−1.8, 0.2) *−0.9%“DV”*	0.10	1.7 (1.2)	−0.1 (−0.3, 0.1)| *−0.4%DV*	0.21	376.4 (319.3)	−13.4 (−56.2, 29.3)| −*0.6%DV*	0.54
55+ yo	457	4.1 (2.4)	−0.3 (−0.7, 0.1) *−1.5%DV*	0.10	9.7 (7.2)	−0.1 (−1.1, 0.9) *−0.1%“DV”*	0.87	1.4 (0.9)	**−0.4** **(−0.6, −0.3)** ***−1.4%DV***	**<0.001**	294.8 (255.2)	**−95.0** **(−137.0, −53.0)** ***−4.1%DV***	**<0.001**
Region													
Northeast (ref)	211	4.6 (2.8)			10.3 (7.2)			1.8 (1.3)			410.5 (331.8)		
South	439	4.1 (2.6)	**−0.5** **(−1.0, −0.1)** ***−2.5%DV***	**0.02**	9.4 (6.8)	−0.9 (−2.1, 0.2) *−1.0%“DV”*	0.12	1.5 (1.1)	**−0.3** **(−0.5, −0.1)** **−*1.1%DV***	**0.001**	326.6 (291.2)	**−84.0** **(−133.7, −34.2)** **−*3.7%DV***	**0.001**
Midwest	293	4.2 (2.4)	−0.4 (−0.9, 0.0) *−2.0%DV*	0.07	9.5 (7.0)	−0.8 (−2.0, 0.5) *−0.9%“DV”*	0.22	1.5 (1.0)	**−0.3** **(−0.5, −0.1)** **−*1.1%DV***	**0.003**	329.3 (268.5)	**−81.2** **(−134.8, −27.6)** **−*3.5%DV***	**0.003**
West	285	4.3 (3.0)	−0.3 (−0.7, 0.2) *−1.5%DV*	0.30	9.2 (7.2)	−1.1 (−2.3, 0.2) *−1.2%“DV”*	0.09	1.6 (1.2)	**−0.3** **(−0.5, −0.1)** **−*1.1%DV***	**0.01**	361.8 (329.3)	−48.8 (−102.7, 5.1) *−2.1%DV*	0.08

(**A**) ^a^ Percentage calculated against a 2000 kcal/day diet. ^b^ Percentage calculated against the daily value for protein of 50 g/day. ^c^ Percentage calculated against the daily value for total fat of 78 g/day. ^d^ Percentage calculated against the daily value for total carbohydrates of 275 g/day. (**B**) ^a^ Percentage calculated against the daily value for saturated fat of 20 g/day. ^b^ Percentage calculated against the United Kingdom’s National Health Service reference intake for total sugars of 90 g/day for adults. ^c^ Percentage calculated against the daily value for dietary fiber of 28 g/day. ^d^ Percentage calculated against the daily value for sodium of 2300 mg/day.

## Data Availability

The original data presented in the study are openly available in the study’s OSF page at https://osf.io/c53ab/overview, accessed 17 September 2025.

## Supplementary Materials

The following supporting information can be downloaded at: https://www.mdpi.com/article/10.3390/nu18193257/s1, Supplemental Figure S1: Eating in America Study Respondent Flow Diagram Towards Nutrition-related Analyses of Comfort Foods; Supplemental Table S1. The Top 10^a^ Comfort Food Groupings (collapsed, then followed by uncollapsed constituent foods) Overall in the US: Prevalence and Nutritional Information, including Hyperpalatability and Nova Group Processing; Supplemental Table S2. Top Comfort Foods^a^ (collapsed) in the US, continued (#51 onwards): Individual and Cumulative Prevalences; Supplemental Table S3. Frequency Matrix of Number of Foods Fitting % Daily Values (per serving), Hyperpalatable Food Types, and Ultra-Processed Foods Amongst Unique Top Comfort Foods (*n* = 300, uncollapsed) in the US—*n* (%); Supplemental Table S4. Unique Comfort Foods’^a^ Likelihood Ratios of Being Hyperpalatable or Ultra-Processed Food Status by Nutrient; Supplemental Table S5. Comparisons of Person-Level Mean Number of Hyperpalatable Foods (by Type) and Ultra-Processed Foods Nominated for Top Comfort Foods by Gender, Age, and US Region of Residence.
