# Supplementary material for: The Nutrition Facts of Comfort Foods in the United States: Results from the Census-Matched Eating in America Study
Source: Nutrients. 2026 Oct 2;18(19):3257. doi: 10.3390/nu18193257 (PMC13667082; doi:10.3390/nu18193257)
Supplement: Supplementary file 1 [file nutrients-18-03257-s001.zip › nutrients-4523095-supplementary.pdf]

Supplemental Figure S1: Eating in America Study Respondent Flow Diagram Towards Nutrition-related Analyses of Comfort Foods

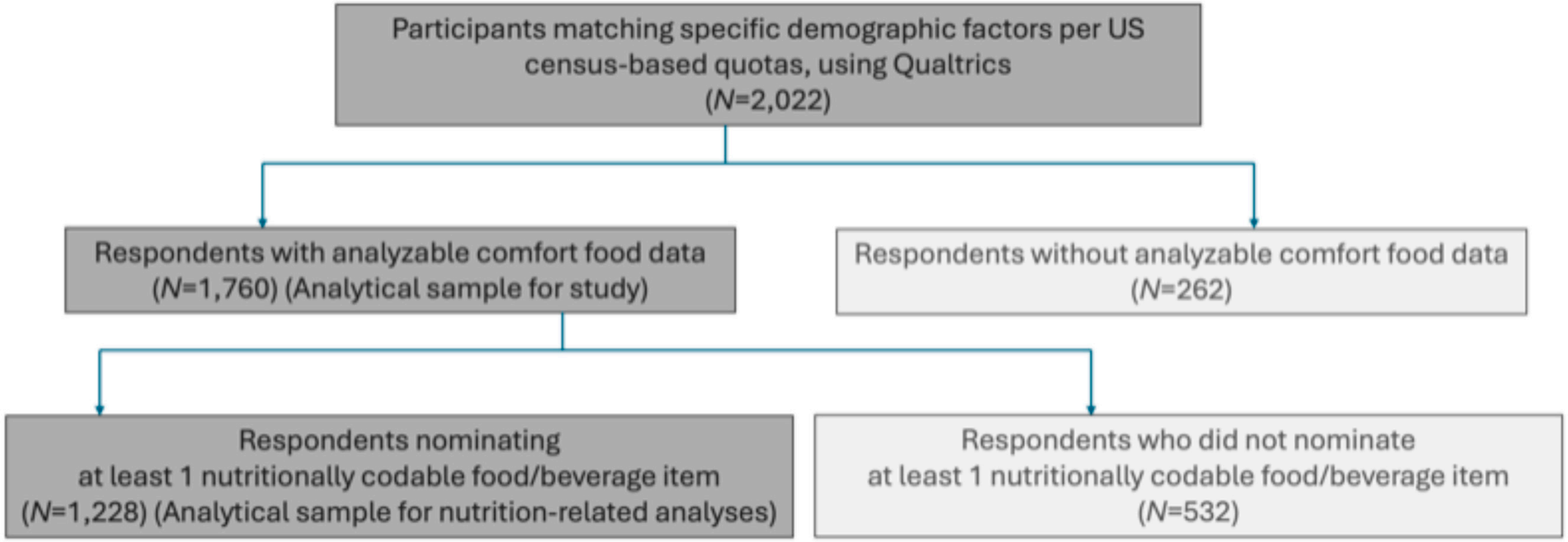

Supplemental Table S1. The Top 10[a] Comfort Food Groupings (collapsed, then followed by uncollapsed constituent foods) Overall in the US: Prevalence and Nutritional Information, including Hyperpalatability and Nova Group Processing

Rank	Collapsed Food	Constituent Food (if applicable)[b]	Prevalence – *n* (%)[c]	Cumulative Prevalence (%)[d]	Measure (Serving) Weight (g)	Total Kcal per serving	Total Protein (g) per serving [e]	Total Fat (g) per serving [e]	Saturated Fat (g) per serving [e]	Total Carbohydrate (g) per serving [e]	Total Sugars (g) per serving [e]	Dietary Fiber (g) per serving [e]	Sodium (mg) per serving [e]	High Fat, High Sugar dessert type[f] Hyperpalatable Food	High Fat, High Sodium savory, meal-type[f] Hyperpalatable Food	High Carb, High Sodium bread/carbohydrate-type[f] Hyperpalatable Food	Proportion[g] of calories in NOVA Group 4
1	Chips (all types, incl. cheese curls/puffs)		425 (13.11%)	13.11%													
		Potato chips, general	395 (12.19%)		28	149.0	*1.8*	9.5	*1.0*	15.1	*0.1*	*0.9*	147.6		Y	Y	1.0
		Potato chips, barbecue	1 (0.03%)		28	136.4	*1.8*	8.7	1.1	15.7	*1.5*	*1.1*	152.6		Y	Y	1.0
		Potato chips, other flavor	2 (0.06%)		28	136.4	*1.8*	8.7	1.1	15.7	*1.5*	*1.1*	152.6		Y	Y	1.0
		Corn chips, plain	4 (0.12%)		45	240.8	2.8	14.9	1.9	25.6	*0.1*	1.8	367.7		Y	Y	1.0
		Doritos	3 (0.09%)		30	150.8	*2.2*	7.5	1.1	18.9	*0.6*	1.6	190.1		Y	Y	1.0
		Cheese flavored corn snacks	3 (0.09%)		35	196	*2.1*	12.6	1.9	18.7	*1.1*	*0.5*	329.7		Y	Y	1.0
		Cheetos	7 (0.22%)		35	196	*2.1*	12.6	1.9	18.7	*1.1*	*0.5*	329.7		Y	Y	1.0
		Takis	1 (0.03%)		30	155.7	*2.2*	8.2	1.2	18.2	*0.8*	1.5	207.3		Y	Y	1.0

Rank	Collapsed Food	Constituent Food (if applicable)[b]	Prevalence – *n* (%)[c]	Cumulative Prevalence (%)[d]	Measure (Serving) Weight (g)	Total Kcal per serving	Total Protein (g) per serving [e]	Total Fat (g) per serving [e]	Saturated Fat (g) per serving [e]	Total Carbohydrate (g) per serving [e]	Total Sugars (g) per serving [e]	Dietary Fiber (g) per serving [e]	Sodium (mg) per serving [e]	High Fat, High Sugar dessert type[f] Hyperpalatable Food	High Fat, High Sodium savory, meal-type[f] Hyperpalatable Food	High Carb, High Sodium bread/carbohydrate-type[f] Hyperpalatable Food	Proportion[g] of calories in NOVA Group 4
2	Ice cream (all, incl. frozen yogurt, dairy free)		324 (10.0%)	23.11%													
		Ice cream, general	319 (9.84%)		135	298.4	4.8	15.3	**9.3**	35.6	**30.9**	*1.1*	121.5	Y			1.0
		Ice cream, chocolate	3 (0.09%)		135	291.6	5.1	14.9	**9.2**	38.1	**34.2**	1.6	*102.6*	Y			1.0
		Ice cream, dairy free	1 (0.03%)		165	254.1	*1.3*	14.8	**11.7**	31.0	**30.2**	*0.2*	*75.9*	Y			1.0
		Frozen yogurt	1 (0.03%)		175	222.3	5.3	6.3	**4.1**	37.8	**34.9**	*0*	*110.3*	Y			1.0
3	Pizza (incl. pizza rolls)		274 (8.45%)	31.56%													

Rank	Collapsed Food	Constituent Food (if applicable)[b]	Prevalence – *n* (%)[c]	Cumulative Prevalence (%)[d]	Measure (Serving) Weight (g)	Total Kcal per serving	Total Protein (g) per serving [e]	Total Fat (g) per serving [e]	Saturated Fat (g) per serving [e]	Total Carbohydrate (g) per serving [e]	Total Sugars (g) per serving [e]	Dietary Fiber (g) per serving [e]	Sodium (mg) per serving [e]	High Fat, High Sugar dessert type[f] Hyperpalatable Food	High Fat, High Sodium savory, meal-type[f] Hyperpalatable Food	High Carb, High Sodium bread/carbohydrate-type[f] Hyperpalatable Food	Proportion[g] of calories in NOVA Group 4
		Pizza	273 (8.42%)		238	633.1	**27.1**	**23.1**	**10.6**	**79.3**	8.5	5.5	**1423.2**		Y		1.0
		Pizza rolls	1 (0.03%)		85	293.3	7.8	8.9	2.2	45.4	*3.4*	*1.1*	**536.4**		Y	Y	1.0
4	Chocolate (all, incl. dark)		260 (8.02%)	39.59%													
		Chocolate candy	244 (7.53%)		21	112.4	*1.61*	6.2	3.9	*12.5*	10.8	*0.7*	*16.6*	Y			1.0
		Dark chocolate	2 (0.06%)		21	110.9	*0.95*	7.0	**4.1**	*12.6*	10.3	*1.3*	*4.2*	Y			1.0
		Almond Joy	1 (0.03%)		21	103.5	*1.0*	5.6	**4.3**	*12.4*	9.7	*0.8*	*30.5*	Y			1.0
		Snickers	2 (0.06%)		21	103.1	*1.6*	5.0	1.9	*12.9*	10.6	*0.5*	*50.2*	Y			1.0

Rank	Collapsed Food	Constituent Food (if applicable)[b]	Prevalence – *n* (%)[c]	Cumulative Prevalence (%)[d]	Measure (Serving) Weight (g)	Total Kcal per serving	Total Protein (g) per serving [e]	Total Fat (g) per serving [e]	Saturated Fat (g) per serving [e]	Total Carbohydrate (g) per serving [e]	Total Sugars (g) per serving [e]	Dietary Fiber (g) per serving [e]	Sodium (mg) per serving [e]	High Fat, High Sugar dessert type[f] Hyperpalatable Food	High Fat, High Sodium savory, meal-type[f] Hyperpalatable Food	High Carb, High Sodium bread/carbohydrate-type[f] Hyperpalatable Food	Proportion[g] of calories in NOVA Group 4
		M&M's (chocolate candy w/ candy shell)	4 (0.12%)		21	103.3	*0.9*	4.4	2.8	15.0	13.4	*0.6*	*12.8*	Y			1.0
		M&M's (peanut)	1 (0.03%)		21	108.2	*2.0*	5.5	2.1	*12.7*	10.7	*0.8*	*10.5*	Y			1.0
		Milky Way (Caramel)	1 (0.03%)		52.2	237.3	*1.8*	9.5	**7.1**	35.6	**30.8**	*0*	*59.3*	Y			1.0
		Snickers (Bite Size)	1 (0.03%)		52.7	253.7	3.9	11.7	**4.9**	33.2	**27.3**	1.5	126.9	Y			1.0
		Snow Caps	1 (0.03%)		21	103.2	*0.9*	4.4	2.8	15.0	13.4	*0.6*	*12.9*	Y			1.0
		Chocolate Peanut Butter	2 (0.06%)		21	108.2	*2.2*	6.4	2.3	*11.6*	9.9	*0.8*	*75.0*	Y	Y		1.0

Rank	Collapsed Food	Constituent Food (if applicable)[b]	Prevalence – *n* (%)[c]	Cumulative Prevalence (%)[d]	Measure (Serving) Weight (g)	Total Kcal per serving	Total Protein (g) per serving [e]	Total Fat (g) per serving [e]	Saturated Fat (g) per serving [e]	Total Carbohydrate (g) per serving [e]	Total Sugars (g) per serving [e]	Dietary Fiber (g) per serving [e]	Sodium (mg) per serving [e]	High Fat, High Sugar dessert type[f] Hyperpalatable Food	High Fat, High Sodium savory, meal-type[f] Hyperpalatable Food	High Carb, High Sodium bread/carbohydrate-type[f] Hyperpalatable Food	Proportion[g] of calories in NOVA Group 4
		Cups (Reese's)															
		Chocolate covered cherries	1 (0.03%)		21	76.9	*0.5*	*2.0*	1.2	16.9	15.0	*0.4*	*5.5*	Y			1.0
5	Cookies (all types)		186 (5.74%)	45.33%													
		Cookie Dough (any type, raw)	2 (0.06%)		63	271.5	3.0	14.0	2.7	34.1	**18.4**	*0.6*	337.1	Y	Y		0.43
		Cookies, general not further specified	174 (5.37%)		60	295.2	3.1	14.8	**4.9**	39.2	**19.7**	*1.2*	186.6	Y	Y		1.0

Rank	Collapsed Food	Constituent Food (if applicable)[b]	Prevalence – *n* (%)[c]	Cumulative Prevalence (%)[d]	Measure (Serving) Weight (g)	Total Kcal per serving	Total Protein (g) per serving [e]	Total Fat (g) per serving [e]	Saturated Fat (g) per serving [e]	Total Carbohydrate (g) per serving [e]	Total Sugars (g) per serving [e]	Dietary Fiber (g) per serving [e]	Sodium (mg) per serving [e]	High Fat, High Sugar dessert type[f] Hyperpalatable Food	High Fat, High Sodium savory, meal-type[f] Hyperpalatable Food	High Carb, High Sodium bread/carbohydrate-type[f] Hyperpalatable Food	Proportion[g] of calories in NOVA Group 4
		Chocolate Chip cookie (home or bakery)	4 (0.12%)		60	293.4	3.5	**16.9**	**7.7**	35.6	**24.7**	1.6	168	Y			0.23
		Oreos	6 (0.19%)		42	194.9	*2.2*	8.0	2.4	29.8	17.1	*1.2*	163.0	Y	Y		1.0
6	Candy (all non chocolate)		173 (5.34%)	50.66%													
		Candy, not further specified	160 (4.94%)		14	55.2	*0*	*0.03*	*0*	*13.7*	8.8	*0*	*5.3*				1.0
		Taffy	1 (0.03%)		14	54.6	*0.03*	*0.6*	*0.5*	*12.3*	10.3	*0*	*0.8*				1.0

Rank	Collapsed Food	Constituent Food (if applicable)[b]	Prevalence – *n* (%)[c]	Cumulative Prevalence (%)[d]	Measure (Serving) Weight (g)	Total Kcal per serving	Total Protein (g) per serving [e]	Total Fat (g) per serving [e]	Saturated Fat (g) per serving [e]	Total Carbohydrate (g) per serving [e]	Total Sugars (g) per serving [e]	Dietary Fiber (g) per serving [e]	Sodium (mg) per serving [e]	High Fat, High Sugar dessert type[f] Hyperpalatable Food	High Fat, High Sodium savory, meal-type[f] Hyperpalatable Food	High Carb, High Sodium bread/carbohydrate-type[f] Hyperpalatable Food	Proportion[g] of calories in NOVA Group 4
		Gummy candy	12 (0.37%)		14	55.4	*0*	*0*	*0*	13.9	8.3	*0.01*	*6.2*				1.0
7	Burgers (incl. Veggie)		104 (3.21%)	53.87%													
		Hamburger, Not further specified	88 (2.72%)		145	417.6	**25.4**	**20.9**	**8.0**	30.0	*4.4*	*1.0*	**514.8**		Y		1.0
		Hamburger w/ Pickle, Onion, and Mustard [h]	1 (0.03%)		100[g]	221.0	**13.3**	10.9	**4.1**	16.5	*2.8*	*0.9*	416.9		Y		0.74
		Hamburger, McDonald's	1 (0.030%)		100	263	**13.3**	10.2	3.6	29.6	6.0	1.8	**487**		Y		1.0

**Rank**	**Collapsed Food**	**Constituent Food (if applicable)[b]**	**Prevalence – *n* (%)[c]**	**Cumulative Prevalence (%)[d]**	**Measure (Serving) Weight (g)**	**Total Kcal per serving**	**Total Protein (g) per serving [e]**	**Total Fat (g) per serving [e]**	**Saturated Fat (g) per serving [e]**	**Total Carbohydrate (g) per serving [e]**	**Total Sugars (g) per serving [e]**	**Dietary Fiber (g) per serving [e]**	**Sodium (mg) per serving [e]**	**High Fat, High Sugar dessert type[f] Hyperpalatable Food**	**High Fat, High Sodium savory, meal-type[f] Hyperpalatable Food**	**High Carb, High Sodium bread/carbohydrate-type[f] Hyperpalatable Food**	**Proportion[g] of calories in NOVA Group 4**
		Cheeseburger, Not further specified	13 (0.40%)		165	490.1	**29.6**	**26.8**	**11.4**	30.7	4.8	*1.0*	**732.6**		Y		0.92
		Impossible Burger (Burger King)	1 (0.03%)		285	630.0	**25.0**	**34.0**	**11.0**	**58.0**	12.0	4.0	**1080.0**		Y		1.0
8	Cake (incl. cupcakes)		92 (2.84%)	56.71%													
		Cake or cupcake, not further specified	89 (2.75%)		39	151.7	*1.4*	7.8	2.3	20.6	15.6	*0.9*	135.7	Y	Y		1.0
		Chocolate cake	1 (0.03%)		109	424.0	3.8	**21.9**	**6.5**	**57.6**	**43.6**	2.4	379.3	Y	Y		1.0

Rank	Collapsed Food	Constituent Food (if applicable)[b]	Prevalence – *n* (%)[c]	Cumulative Prevalence (%)[d]	Measure (Serving) Weight (g)	Total Kcal per serving	Total Protein (g) per serving [e]	Total Fat (g) per serving [e]	Saturated Fat (g) per serving [e]	Total Carbohydrate (g) per serving [e]	Total Sugars (g) per serving [e]	Dietary Fiber (g) per serving [e]	Sodium (mg) per serving [e]	High Fat, High Sugar dessert type[f] Hyperpalatable Food	High Fat, High Sodium savory, meal-type[f] Hyperpalatable Food	High Carb, High Sodium bread/carbohydrate-type[f] Hyperpalatable Food	Proportion[g] of calories in NOVA Group 4
		Lemon cake	1 (0.03%)		109	421.8	3.7	**19.1**	**6.3**	**59.9**	**43.0**	*0.3*	**463.3**	Y	Y		0.44
		Tres leches cake	1 (0.03%)		120	320.4	7.62	10.5	**6.0**	50.2	**39.8**	*0.4*	192	Y			0.14
9	Pasta incl. Noodles (unspecified)		88 (2.72%)	59.43%													
		Pasta (with sauce), not further specified	72 (2.22%)		250	307.5	**14.7**	6.5	1.9	47.9	6.1	4.0	**500**				0.16
		Egg noodles with mashed potatoes [h]	1 (0.03%)		100[g]	126.3	3.5	*2.9*	1.2	21.8	*1.2*	*1.2*	225.6				0.02

Rank	Collapsed Food	Constituent Food (if applicable)[b]	Prevalence – *n* (%)[c]	Cumulative Prevalence (%)[d]	Measure (Serving) Weight (g)	Total Kcal per serving	Total Protein (g) per serving [e]	Total Fat (g) per serving [e]	Saturated Fat (g) per serving [e]	Total Carbohydrate (g) per serving [e]	Total Sugars (g) per serving [e]	Dietary Fiber (g) per serving [e]	Sodium (mg) per serving [e]	High Fat, High Sugar dessert type[f] Hyperpalatable Food	High Fat, High Sodium savory, meal-type[f] Hyperpalatable Food	High Carb, High Sodium bread/carbohydrate-type[f] Hyperpalatable Food	Proportion[g] of calories in NOVA Group 4
		Plain pasta/ macaroni	4 (0.12%)		140	219.8	8.1	*1.3*	*0.2*	43.0	*0.8*	2.5	324.8				0
		Tomatoes and mac [h]	1 (0.03%)		100[g]	107.7	3.6	*2.1*	*0.5*	18.7	*1.8*	1.7	198.5				0.04
		Noodles (cooked)	10 (0.31%)		160	219.2	7.2	3.3	*0.7*	40.0	*0.6*	1.9	377.6				0
10	Bread (incl w/ spread)		82 (2.53%)	61.96%													
		Bread, not further specified	68 (2.10%)		28	75.6	2.6	*1.0*	*0.2*	13.8	*1.5*	*0.6*	133.6			Y	1.0
		Bread and Butter [h]	2 (0.06%)		100[g]	419.1	6.6	**29.5**	**17.7**	32.8	*3.6*	1.4	**533.3**		Y		0.67
		Toast	1 (0.03%)		25	74.3	2.6	*1.0*	*0.2*	*13.5*	*1.5*	*0.6*	131			Y	1.0

**Rank**	**Collapsed Food**	**Constituent Food (if applicable)[b]**	**Prevalence – *n* (%)[c]**	**Cumulative Prevalence (%)[d]**	**Measure (Serving) Weight (g)**	**Total Kcal per serving**	**Total Protein (g) per serving [e]**	**Total Fat (g) per serving [e]**	**Saturated Fat (g) per serving [e]**	**Total Carbohydrate (g) per serving [e]**	**Total Sugars (g) per serving [e]**	**Dietary Fiber (g) per serving [e]**	**Sodium (mg) per serving [e]**	**High Fat, High Sugar dessert type[f] Hyperpalatable Food**	**High Fat, High Sodium savory, meal-type[f] Hyperpalatable Food**	**High Carb, High Sodium bread/carbohydrate-type[f] Hyperpalatable Food**	**Proportion[g] of calories in NOVA Group 4**
		Toast w/ peanut butter [h]	1 (0.03%)		100[g]	464.9	**17.2**	**30.4**	**6.1**	36.3	8.5	3.7	**470.2**		Y		0.47
		Toast w/ butter [h]	1 (0.03%)		100[g]	305.4	4.8	**21.7**	**13.0**	23.7	*2.6*	*1.1*	387.7		Y		0.67
		Homemade Bread	1 (0.03%)		44	118.8	3.8	*1.5*	*0.5*	22.0	*1.4*	*0.8*	249.0			Y	0.03
		Roll, Not Specific	2 (0.06%)		43	120.0	4.2	*1.7*	*0.4*	21.6	*3.1*	*0.8*	212.4			Y	1.0
		Garlic bread	1 (0.03%)		79	275.7	6.6	13.1	**4.3**	32.9	*2.9*	2.0	**489.8**		Y		1.0
		Fried bread	1 (0.03%)		26	99.3	*2.1*	4.9	1.4	*11.7*	*0.9*	*0.4*	*45.8*				1.0
		Biscuits	2 (0.06%)		45	166.5	3.2	8.5	**5.3**	19.3	*1.8*	*1.1*	440.6		Y		1.0

Rank	Collapsed Food	Constituent Food (if applicable)[b]	Prevalence – *n* (%)[c]	Cumulative Prevalence (%)[d]	Measure (Serving) Weight (g)	Total Kcal per serving	Total Protein (g) per serving [e]	Total Fat (g) per serving [e]	Saturated Fat (g) per serving [e]	Total Carbohydrate (g) per serving [e]	Total Sugars (g) per serving [e]	Dietary Fiber (g) per serving [e]	Sodium (mg) per serving [e]	High Fat, High Sugar dessert type[f] Hyperpalatable Food	High Fat, High Sodium savory, meal-type[f] Hyperpalatable Food	High Carb, High Sodium bread/carbohydrate-type[f] Hyperpalatable Food	Proportion[g] of calories in NOVA Group 4
		Cornbread	2 (0.06%)		60	198	4.0	5.8	2.2	32.7	9.6	*1.4*	359.4		Y		1.0

[a] Including ties.
[b] Food name is a summary of the FNDDS label for the items when it was nominated in more than 1 way. When item was named once, food name was derived from survey respondent's terms as much as possible.
[c] Out of non-missing (non-blank), codable items where *n*=3,241.
[d] Only for collapsed foods.
[e] Italicized entries reflect a food's status as a low source of said nutrient based on a 2,000 kcal diet. Bold entries reflect a food's status as a high source of said nutrient based on a 2,000 kcal diet. Low sources of fiber and high sources of saturated fat, total sugars, and sodium were shaded red. Low sources of saturated fat, total sugars, and sodium were shaded green.
[f] Y denotes "yes", this food item fits the criteria of the category. Blank means it does not.
[g] Foods where the whole proportion (1.0) of calories were attributable to NOVA group 4 foods were considered ultra-processed foods (UPFs).
[h] These foods were calculated to be 100g to facilitate comparisons and this value should not be taken as a typical serving size. As these are combination foods, it is unknown how this per-100g value would relate to one's actual "serving" of the combination item.

Supplemental Table S2. Top Comfort Foods[a] (collapsed) in the US, continued (#51 onwards): Individual and Cumulative Prevalences

Rank	Food	Prevalence *n* (%)	Cumulative Prevalence (%)
52	Chicken nuggets/nuggets	6 (0.2%)	93.4%
52	Beans	6 (0.2%)	93.6%
52	Burritos (incl. frozen)	6 (0.2%)	93.8%
52	Apples	6 (0.2%)	94.0%
52	Shellfish/shellfish dishes (incl. octopus)	6 (0.2%)	94.1%
52	Bagels (with or without toppings)	6 (0.2%)	94.3%
58	Yogurt	5 (0.2%)	94.5%
58	Pop Tarts	5 (0.2%)	94.6%
58	Pickles	5 (0.2%)	94.8%
58	Sugar	5 (0.2%)	94.9%
58	Roast beef/pot roast	5 (0.2%)	95.1%
58	Bacon, sausage	5 (0.2%)	95.3%
58	Sweet breakfast foods (e.g. pancakes, French toast, waffles)	5 (0.2%)	95.4%
65	Milk	4 (0.1%)	95.5%
65	Gravy/gravy sauce	4 (0.1%)	95.7%
65	Lasagna	4 (0.1%)	95.8%
65	Sushi	4 (0.1%)	95.9%
65	Wine	4 (0.1%)	96.0%
65	Beer	4 (0.1%)	96.1%
65	Water/ice	4 (0.1%)	96.3%
65	Ice cream desserts (e.g. hot fudge sundae, banana split, milkshake, root beer float)	4 (0.1%)	96.4%
65	Meat/chicken dish and potato combinations	4 (0.1%)	96.5%
65	Trail mix/GORP	4 (0.1%)	96.6%
65	Snack cakes/Twinkies	4 (0.1%)	96.8%
65	Granola/breakfast bars	4 (0.1%)	96.9%
65	Alfredo dishes (chicken, shrimp, fettucine)	4 (0.1%)	97.0%
78	Cocoa/hot cocoa	3 (0.09%)	97.1%
78	Beef jerky/Slim Jims	3 (0.09%)	97.2%
78	Oatmeal/oats	3 (0.09%)	97.3%
78	Grapes	3 (0.09%)	97.4%
78	Juice/fruit juice (not specified as 100%)	3 (0.09%)	97.5%
78	Onion rings	3 (0.09%)	97.6%
78	Diet Cola (diet Coke/diet Pepsi)	3 (0.09%)	97.7%
78	Cracker combinations (e.g. crackers + cheese, peanut butter, or lunch meat and cheese)	3 (0.09%)	97.8%
78	Ham/pork chops	3 (0.09%)	97.8%
78	Lunch meat, olive loaf, cold cuts	3 (0.09%)	97.9%

78	TV/frozen dinners, turkey dinners	3 (0.09%)	98.0%
78	Muffins (incl. corn)	3 (0.09%)	98.1%
78	Yams, candy yams	3 (0.09%)	98.2%
91	Mozzarella sticks, fried cheese sticks	2 (0.06%)	98.3%
91	Turkey	2 (0.06%)	98.3%
91	Stuffing	2 (0.06%)	98.4%
91	Bananas	2 (0.06%)	98.5%
91	Mangos	2 (0.06%)	98.5%
91	Broccoli	2 (0.06%)	98.6%
91	Olives	2 (0.06%)	98.6%
91	Butter	2 (0.06%)	98.7%
91	Jello	2 (0.06%)	98.8%
91	Twizzlers/licorice	2 (0.06%)	98.8%
91	Pudding (incl. chocolate pudding)	2 (0.06%)	98.9%
91	Tiramisu, crème brulee	2 (0.06%)	99.0%
91	Pork rinds/chicharrones	2 (0.06%)	99.0%
91	Ham/bacon and eggs	2 (0.06%)	99.1%
91	Soup and sandwich/sandwich-like dish combinations (e.g., tomato soup + grilled cheese, soup + quesadilla)	2 (0.06%)	99.1%
91	Potstickers/dumplings	2 (0.06%)	99.2%
107	Gizzards	1 (0.03%)	99.2%
107	American chop suey	1 (0.03%)	99.3%
107	Croutons	1 (0.03%)	99.3%
107	S'mores	1 (0.03%)	99.3%
107	Grits	1 (0.03%)	99.4%
107	Quesadillas	1 (0.03%)	99.4%
107	Taquitos	1 (0.03%)	99.4%
107	Pad thai	1 (0.03%)	99.4%
107	Dried cranberries	1 (0.03%)	99.5%
107	Apricots	1 (0.03%)	99.5%
107	Potato skins	1 (0.03%)	99.5%
107	Tater tots	1 (0.03%)	99.6%
107	Carrots	1 (0.03%)	99.6%
107	Salsa	1 (0.03%)	99.6%
107	Onions	1 (0.03%)	99.7%
107	String beans	1 (0.03%)	99.7%
107	Peas	1 (0.03%)	99.7%
107	Cheese-stuffed jalapeño peppers	1 (0.03%)	99.8%
107	Fried okra	1 (0.03%)	99.8%
107	Hot sauce	1 (0.03%)	99.8%
107	Curry	1 (0.03%)	99.9%
107	Pierogies	1 (0.03%)	99.9%
107	Nutella	1 (0.03%)	99.9%

107	Jelly	1 (0.03%)	99.9%
107	Peanut brittle	1 (0.03%)	100%
107	Energy drinks	1 (0.03%)	100%

[a] As similar foods were grouped together where possible from research team discussions, this list could be better considered as a list of top comfort food *grouping*s in the United States. A grouping can comprise of many nominations of a type of food or beverage, or just one (e.g., for chips, many items that were nutritionally disparate were collapsible into a "chip" category given similarities perceived by the research team, but for pierogies, there was only 1 nomination and no other food deemed similar so it comprised its own grouping).

Supplemental Table S3. Frequency Matrix of Number of Foods Fitting % Daily Values (per serving), Hyperpalatable Food Types, and Ultra-Processed Foods Amongst Unique Top Comfort Foods (*n*=300, uncollapsed) in the US – *n* (%)

	Low Protein	High Fat	High Carbohydrate	High Sugar	*Low Sugar*	Low Dietary Fiber	High Saturated Fat	High Sodium	High Fat, High Sugar ("dessert")	High Fat, High Sodium ("meats/meal")	High Carbohydrate, High Sodium ("bread/carb")	Ultra-Processed Food[a]
% Daily Values												
Low Protein	/											
High Fat	4 (1.3%)	/										
High Carbohydrate	0 (0.0%)	11 (3.7%)	/									
High Sugar	17 (5.7%)	15 (5.0%)	7 (2.3%)	/								
Low Sugar	47 (15.7%)	32 (10.7%)	5 (1.7%)	0	/							
Low Dietary Fiber	77 (25.7%)	26 (8.7%)	1 (0.3%)	30 (10.0%)	115 (38.3%)	/						
High Saturated Fat	14 (4.7%)	57 (19.0%)	9 (3.0%)	25 (8.3%)	37 (12.3%)	46 (15.3%)	/					
High Sodium	1 (0.3%)	44 (14.7%)	10 (3.3%)	3 (1.0%)	58 (19.3%)	41 (13.7%)	44 (14.7%)	/				
Hyperpalatable Food type												
High Fat, High Sugar ("dessert")	25 (8.3%)	13 (4.3%)	6 (2.0%)	38 (12.7%)	2 (0.7%)	42 (14.0%)	29 (9.7%)	3 (1.0%)	/			
High Fat, High Sodium ("meats/meal")	33 (11.0%)	53 (17.7%)	9 (3.0%)	14 (4.7%)	111 (37.0%)	104 (34.7%)	55 (18.3%)	76 (25.3%)	20 (6.7%)	/		
High Carbohydrate, High Sodium ("bread/carb")	19 (6.3%)	1 (0.3%)	3 (1.0%)	1 (0.3%)	28 (9.3%)	22 (7.3%)	1 (0.3%)	2 (0.7%)	22 (7.3%)	0 (0.0%)	/	
NOVA Processing												
Ultra-Processed Food[a]	57 (19.0%)	24 (8.0%)	10 (3.3%)	34 (11.3%)	54 (18.0%)	82 (27.3%)	39 (13.0%)	37 (12.3%)	70 (23.3%)	44 (14.7%)	31 (10.3%)	/

[a] Where the proportion of calories from Nova Group 4 is 1.0 or 100%.

Supplemental Table S4. Unique Comfort Foods'[a] Likelihood Ratios of Being Hyperpalatable or Ultra-Processed Food Status by Nutrient

	High fat, high sugar "dessert"-type hyperpalatable food		High fat, high sodium "meats/ meal"-type hyperpalatable food		High carbohydrate, high sodium "bread/ carbohydrate"-type hyperpalatable food		Ultra-Processed Food[b]	
Nutrient	Likelihood Ratio (95% CI)	p-value	Likelihood Ratio (95% CI)	p-value	Likelihood Ratio (95% CI)	p-value	Likelihood Ratio (95% CI)	p-value
Calories (kcal)	1.00 (1.00, 1.00)	0.117	**1.001 (1.000, 1.002)**	**0.001**	**0.997 (0.994, 0.999)**	**0.01**	1.000 (0.999, 1.001)	0.64
Protein (g)	**0.91 (0.86, 0.97)**	**0.002**	**1.03 (1.02, 1.04)**	**<0.001**	**0.86 (0.80, 0.92)**	**<0.001**	**0.95 (0.93, 0.97)**	**<0.001**
Total Fat (g)	1.01 (0.99, 1.03)	0.43	**1.03 (1.02, 1.04)**	**<0.001**	**0.93 (0.89, 0.97)**	**0.001**	0.99 (0.98, 1.01)	0.40
Carbohydrate (g)	**1.02 (1.01, 1.02)**	**<0.001**	**0.99 (0.99,1.00)**	**0.03**	1.00 (0.99, 1.02)	0.79	**1.01 (1.01, 1.02)**	**<0.001**
Sugar (g)	**1.03 (1.02, 1.05)**	**<0.001**	**0.97 (0.95, 0.98)**	**<0.001**	**0.91 (0.84, 0.96)**	**0.02**	**1.02 (1.01, 1.02)**	**<0.001**
Dietary Fiber (g)	0.86 (0.74, 1.01)	0.06	0.99 (0.92, 1.06)	0.76	0.92 (0.72, 1.18)	0.51	0.96 (0.88, 1.06)	0.41
Saturated Fat (g)	**1.07 (1.04, 1.11)**	**<0.001**	**1.04 (1.01, 1.06)**	**0.01**	**0.62 (0.51, 0.75)**	**<0.001**	0.99 (0.95, 1.02)	0.47
Sodium (mg)	**0.997 (0.996, 0.998)**	**<0.001**	**1.00 (1.00, 1.00)**	**<0.001**	**0.999 (0.997, 1.000)**	**0.004**	1.00 (1.00, 1.00)	0.47

[a] For all unique nutritionally codable comfort foods (*n*=300).

[a] Foods where the proportion of calories from NOVA Group 4 is 1.0 or 100%.

Supplemental Table S5. Comparisons of Person-Level Mean Number of Hyperpalatable Foods (by Type) and Ultra-Processed Foods Nominated for Top Comfort Foods by Gender, Age, and US Region of Residence

			High fat, high sugar “dessert”-type HPF foods			High fat, high sodium “meats/meal”-type HPF foods			High carbohydrate, high sodium “bread/carb”-type HPF foods			Ultra-Processed Food[a]	
Demographic Characteristic	*n*	Mean[b] (SD)	Difference (95% CI)	p-value	Mean[b] (SD)	Difference (95% CI)	p-value	Mean[b] (SD)	Difference (95% CI) | %DV	p-value	Mean[b] (SD)	Difference (95% CI)	p-value
Gender													
Woman (ref)	649	0.35 (0.33)			0.51 (0.31)			0.21 (0.25)			0.77 (0.30)		
Man	577	0.25 (0.30)	**-0.10 (-0.14, -0.07)**	**<0.001**	0.60 (0.32)	**0.08 (0.05, 0.12)**	**<0.001**	0.17 (0.24)	**-0.04 (-0.07, -0.02)**	**0.002**	0.68 (0.34)	**-0.09 (-0.12, -0.05)**	**<0.001**
Age													
18-34 yo (ref)	349	0.28 (0.31)			0.56 (0.29)			0.19 (0.25)			0.75 (0.31)		
35-54 yo	422	0.27 (0.32)	-0.01 (-0.05, 0.04)	0.75	0.54 (0.33)	-0.02 (-0.06, 0.03)	0.46	0.17 (0.23)	-0.02 (-0.05, 0.02)	0.29	0.70 (0.33)	**-0.05 (-0.09, -0.00)**	**0.04**
55+ yo	457	0.34 (0.33)	**0.06 (0.02, 0.11)**	**0.008**	0.56 (0.31)	-0.00 (-0.04, 0.04)	0.96	0.21 (0.25)	0.02 (-0.01, 0.06)	0.21	0.73 (0.32)	-0.02 (-0.06, 0.03)	0.48
Region													
Northeast (ref)	211	0.30 (0.31)			0.54 (0.30)			0.15 (0.22)			0.70 (0.33)		
South	439	0.31 (0.33)	0.01 (-0.04, 0.07)	0.60	0.54 (0.31)	0.01 (-0.04, 0.06)	0.79	0.19 (0.24)	**0.04 (0.00, 0.08)**	**0.04**	0.73 (0.32)	0.03 (-0.02, 0.08)	0.27
Midwest	293	0.30 (0.31)	-0.00 (-0.06, 0.06)	0.96	0.55 (0.31)	0.01 (-0.05, 0.07)	0.71	0.21 (0.24)	**0.06 (0.01, 0.10)**	**0.01**	0.73 (0.33)	0.03 (-0.03, 0.09)	0.30
West	285	0.28 (0.33)	-0.02 (-0.08, 0.04)	0.52	0.58 (0.32)	0.04 (-0.01, 0.10)	0.13	0.20 (0.26)	**0.05 (0.01, 0.09)**	**0.03**	0.73 (0.31)	0.02 (-0.03, 0.08)	0.42

[a] Where the proportion of calories from Nova Group 4 is 1.0 or 100%.
[b] Where values closer to 1 denote more of said type of foods present amongst person’s top comfort foods.
